# Additions to the knowledge of *Ganoderma* in Thailand: *Ganoderma casuarinicola*, a new record; and *Ganoderma thailandicum* sp. nov.

**DOI:** 10.3897/mycokeys.59.36823

**Published:** 2019-10-16

**Authors:** Thatsanee Luangharn, Samantha C. Karunarathna, Peter E. Mortimer, Kevin D. Hyde, Jianchu Xu

**Affiliations:** 1 Key Laboratory for Plant Diversity and Biogeography of East Asia, Kunming Institute of Botany, Chinese Academy of Sciences, Kunming 650201, Yunnan, China; 2 University of Chinese Academy of Sciences, Beijing 100049, China; 3 East and Central Asia Regional Office, World Agroforestry Centre (ICRAF), Kunming 650201, Yunnan, China; 4 Centre for Mountain Futures (CMF), Kunming Institute of Botany, Kunming 650201, Yunnan, China; 5 Center of Excellence in Fungal Research, Mae Fah Luang University, Chiang Rai 57100, Thailand

**Keywords:** Ganodermataceae, medicinal mushroom, molecular phylogeny, morphological characteristics, new species, white rot

## Abstract

*Ganoderma* is a cosmopolitan genus of mushrooms, which can cause root and butt rot diseases on many tree species. Members of this genus are particularly diverse in tropical regions. Some *Ganoderma* spp. are medicinally active and therefore are used to treat human diseases or as a dietary supplement. In this study, three *Ganoderma* strains were collected in tropical southern Thailand. Phylogenetic analyses of combined ITS, LSU, TEF1α and RPB2 sequence data indicated that the three strains grouped in a distinct lineage within laccate *Ganoderma*. One strain was collected from Surat Thani Province clustered in the *G. casuarinicola* clade with high statistical support (MLBS = 100% / MPBS = 98% / PP = 0.96), while the other two strains of *Ganoderma*, collected from Nakhon Si Thammarat Province, formed a distinct well-supported clade (MLBS = 100% / MPBS = 100% / PP = 1.00) and are described here as a new species. *Ganoderma casuarinicola* is reported here as a new record to Thailand. Morphological differences of the two taxa and their closely related taxa are discussed. Colour photographs of macro and micro morphological characteristics and a phylogenetic tree to show the placement of the new record and new species are provided.

## Introduction

*Ganoderma*, a genus of the Ganodermataceae, was established by Karsten (1881) with *G. lucidum* (Curtis) P. Karst. as the type species. Justo et al. (2017) treated Ganodermataceae as a synonym of Polyporaceae, while Cui et al. (2019) state that *Ganoderma* was not included in Polyporaceae because their double-walled basidiospores are quite different from Polyporaceae. Relevant characteristics for *Ganoderma* species delimitation are unique to laccate and non-laccate basidiocarps: truncated double walled basidiospores, an apical germinal pore, a thin and colourless external wall (exosporium) and a dark brown internal wall (endosporium) (Moncalvo and Ryvarden 1997; Zhao 1989; Núñez and Ryvarden 2000; Ryvarden 2004). *Ganoderma* is a cosmopolitan genus and some of the species are pathogenic, causing white rot diseases on rotting stumps, roots and living trunks (Moncalvo and Ryvarden 1997; Pilotti et al. 2004). *Ganoderma* are distributed in both tropical and temperate regions, but are particularly diverse in the tropical regions (Cao and Yuan 2013). Index Fungorum records 451 taxa (http://www.indexfungorum.org/; accessed date: 1 June 2019) and MycoBank records 387 taxa (http://www.mycobank.org/; accessed date: 1 June 2019). *Ganoderma* can be a confusing genus to study due to the highly variable morphological features of the species in this group, including intra-species variations (Ryvarden 2000; Papp et al. 2017; Hapuarachchi et al. 2018a, c; Hapuarachchi et al. 2019a, b).

The genus *Ganoderma* is economically important, as the members of the genus are regarded as valuable medicinal mushrooms (Dai et al. 2009; Hapuarachchi et al. 2018b). *Ganoderma* spp. have been used in traditional medicines for hundreds of years in Asian countries. Several *Ganoderma* species are known to be prolific sources of highly active bioactive compounds such as polysaccharides, proteins, steroids and triterpenoids, such as ganoderic acids (Shim et al. 2004; Qiao et al. 2005; Wang and Liu 2008; Teng et al. 2011; De Silva et al. 2012a, b; De Silva et al. 2013; Li et al. 2018). Those bioactive compounds have a therapeutic potential to treat and remedy many pathological diseases (Sanodiya et al. 2009; Richter et al. 2015; Hapuarachchi et al. 2018b).

Most members of *Ganoderma* are regarded as plant pathogens for trees, such as *G. australe* (Jungh.) Bres., which is associated with *Castanopsis* spp. (Luangharn et al. 2017); *G. boninense* Pat., which is the causal agent of oil palm basal stem rot (Pilotti 2005); *G. dunense*, which is associated with *Acacia cyclops* (Tchoumi et al. 2018); *G. leucocontextum* T.H. Li, W.Q. Deng, Sheng H. Wu, Dong M. Wang & H.P. Hu, which causes problems to *Cyclobalanopsis glauca* (Li et al. 2015); *G. philippii* (Bres. & Henn. ex Sacc.) Bres., which causes problems to tea and rubber (Zakaria et al. 2009); *G. tropicum*, which grows in a solitary manner on living *Dipterocarpus* spp. (Luangharn et al. 2019); and the holotype of *G. casuarinicola*, which was found associated with a living *Casuarina equisetifolia* tree (Xing et al. 2018).

In Thailand, several *Ganoderma* species have been reported based on both morphological characteristics and molecular data, including *G. australe* (Luangharn et al. 2017), *G. sichuanense* (Thawthong et al. 2017) and *G. tropicum* (Luangharn et al. 2019). The aims of the present study are to report *G. casuarinicola* as a new record to Thailand and describe *G. thailandicum* as a new species from Thailand, based on both morphological characteristics and phylogenetic data.

## Methods

### Mushroom collections and morphological study

Three specimens of *Ganoderma* were photographed at the collecting sites: one from a tropical climate at Surat Thani Province and the other two from Prachuap Khiri Khan Province in Thailand during the rainy season. The detailed morphological characteristics of the specimens were recorded, based on fresh materials (Luangharn et al. 2017). Specimens were subsequently dried at 40 °C for 24 hours, covered with wax papers, kept in sealed plastic bags with anhydrous silica gel (Luangharn et al. 2017) and deposited in the Mae Fah Luang University herbarium (MFLU herb.), while being duplicated in the Herbarium of Cryptogams, Kunming Institute of Botany Academia Sinica (HKAS).

Morphological characteristics were determined following the methodology described by Lodge et al. (2004). Colour changes on bruising were recorded in the field. Colours were recorded following Ridgeway (Ridgeway 1912). Micro-morphological characteristics were observed using a compound Carl Zeiss™ SteREO Discovery.V8 Microscope, while basidiospores were photographed using a Scanning Electron Microscope (SEM). Microscopic features and measurements were made from glass slide preparations, staining the tissues with 3–5% potassium hydroxide (KOH), 2% Melzer’s reagent and 3% Congo red reagent (Kreisel and Schauer 1987). Measurements were made using the Tarosoft Image Framework programme v. 0.9.0.7. Basidiospore features, hyphal system, colour, sizes and shapes were recorded and photographed. The description of basidiospore measurements was done by using at least 50 basidiospores from each basidiomata (Miettinen and Larsson 2006). The basidiospore quotient was followed [*Q = L/W*], where Q, the quotient of basidiospore length to width (L/W) of a basidiospore in side view and Qm, the mean of Q-values ± SD, was calculated considering the mean value of the lengths and widths of basidiospores (Tulloss 2005). The basidiospore size was measured with and without the myxosporium and given as (*a*–)*b*–*c* (–*d*) (Tulloss 2005).

### DNA extraction, PCR amplification and sequencing

Dried internal tissues of the fruiting bodies were used to extract DNA by using the Biospin Fungus Genomic DNA Extraction Kit (BioFlux), following the manufacturer’s instructions. Total reaction mixtures (25 μl) contained 9.5 μl ddH_2_O, 12.5 μl of PCR master mix, 1 μl of DNA template and 1 μl of each primer (10 μM). The primers used in PCR amplification were: ITS4/ITS5 for internal transcribed spacer gene region (ITS); LROR/LR5 for partial large subunit rDNA gene region (LSU) (Vilgalys and Hester 1990; White et al. 1990); 983F/2218R for partial translation elongation factor 1-alpha gene region (TEF1α) (Sung et al. 2007); and fRPB2-5f/fRPB2-7cR for partial RNA polymerase II second largest subunit gene (RPB2) (Liu et al. 1999). PCR amplification conditions were 3 min at 94 °C, followed by 35 cycles of 95 °C for 30 s, 55 °C for 1 min, 72 °C for 1 min, followed by a final extension at 72 °C for 10 min for ITS and LSU. The amplification condition for TEF1α consisted of initial denaturation at 5.30 min at 95 °C, followed by 35 cycles of 94 °C for 1 min, 57 °C for 30 s and 72 °C for 1.30 min, followed by a final extension at 72 °C for 10 min and 3 min at 94 °C followed by 35 cycles of 95 °C for 1 min, 52 °C for 2 min and 72 °C for 1 min, followed by a final extension at 72 °C for 10 min for RPB2. PCR products were sequenced by Sangon Biotech (Shanghai) Co., Ltd., Shanghai, China.

### Phylogenetic analyses

Sequence data, retrieved from GenBank based on previous studies, are listed in Table 1. The sequences were subjected to standard BLAST searches in GenBank to determine the primary identity of the fungal isolates. *Amauroderma rugosum* Cui 9011 (Li and Yuan 2015) and *Tomophagus colossus* (Zhou et al. 2015) were selected as the outgroup taxa. All the newly generated sequences were aligned with the combined datasets of ITS, LSU and TEF1α with MAFFT v. 7.309 (Katoh and Standley 2013) and manually adjusted using Bioedit v. 7.2.5 (Hall 1999). Gaps were treated as missing data. Maximum parsimony (MP) analysis was performed with PAUP v. 4.0b10 (Swofford 2002). Maximum likelihood analyses (ML) were estimated by using the software on the CIPRES Gateway platform (Miller et al. 2010) and performed using RAxML-HPC2 on XSEDE (v. 8.2.8) (Stamatakis 2014), then carried out using the raxmlGUI version v. 1.3.1 (Silvestro and Michalak 2011).

**Table 1.** Details of the taxa used in the phylogenetic analysis of this study. The newly generated sequences are in bold.

Fungal species	Voucher	GenBank accession no.				References
		ITS	LSU	TEF1α	RPB2	
*Ganoderma angustisporum*	Cui 13817	MG279170	–	MG367563	MG367507	Xing et al. 2018
*G. angustisporum*	Cui 14578	MG279171	–	MG367564	–	Xing et al. 2018
*G. aridicola*	Dai 12588	KU572491	–	KU572502	–	Xing et al. 2016
*G. boninense*	WD 2028	KJ143905	KU220015	KJ143924	KJ143964	Zhou et al. 2015
	WD 2085	KJ143906	–	KJ143925	KJ143965	Zhou et al. 2015
*G. carocalcareus*	DMC 322	EU089969	–	–	–	Douanla-Meli and Langer 2009
	DMC 513	EU089970	–	–	–	Douanla-Meli and Langer 2009
*G. casuarinicola*	Dai16336	MG279173	–	MG367565	MG367508	Xing et al. 2018
	Dai16337	MG279174	–	MG367566	MG367509	Xing et al. 2018
	Dai16338	MG279175	–	MG367567	MG367510	Xing et al. 2018
	Dai16339	MG279176	–	MG367568	MG367511	Xing et al. 2018
***G. casuarinicola***	**HKAS 104639**	**MK817650**	**MK817654**	**MK871328**	**MK840868**	**This study**
***G. thailandicum***	**HKAS 104640 (holotype)**	**MK848681**	**MK849879**	**MK875829**	**MK875831**	**This study**
	**HKAS 104641 (paratype)**	**MK848682**	**MK849880**	**MK875830**	**MK875832**	**This study**
*G. ecuadoriense*	ASL799	KU128524	KX228350	–	–	Crous et al. 2016
	PMC126	KU128525	KU128529	–	–	Crous et al. 2016
*G. enigmaticum*	Dai 15970	KU572486	–	KU572496	MG367513	Xing et al. 2016
	Dai 15971	KU572487	–	KU572497	MG367514	Xing et al. 2016
*G. heohnelianum*	Dai 11995	KU219988	–	MG367550	MG367497	Song et al. 2016
	Yuan 6337	MG279160	–	MG367551	MG367498	Xing et al. 2018
	Cui 13982	MG279178	–	MG367570	MG367515	Xing et al. 2018
*G. leucocontextum*	Dai 15601	–	–	–	MG367516	Xing et al. 2016
	GDGM 44303	KJ027607	–	–	–	Li et al. 2015
	GDGM 40200	KF011548	–	–	–	Li et al. 2015
*G. lobatum*	JV 1008/31	KF605671	–	MG367553	MG367499	Xing et al. 2018
	JV 1008/32	KF605670	–	MG367554	MG367500	Xing et al. 2018
*G. lucidum*	K175217	KJ143911	–	KJ143929	KJ143971	Zhou et al. 2015
	Cui 14404	MG279181	–	MG367573	MG367519	Xing et al. 2018
	Cui 14405	MG279182	–	MG367574	MG367520	Xing et al. 2018
*G. lingzhi*	Wu1006-38	JQ781858	–	JX029976	JX029980	Cao et al. 2012
	Cui 14342	MG279179	–	MG367571	MG367517	Xing et al. 2018
*G. martinicense*	LIP SWMart 08-55	KF963256	–	–	–	Welti and Courtecuisse 2010
	LIP SWMart 08-44	KF963257	–	–	–	Welti and Courtecuisse 2010
*G. mbrekobenum*	UMN7-3 GHA	KX000896	KX000897	–	–	Crous et al. 2016
	UMN7-4 GHA	KX000898	KX000899	–	–	Crous et al. 2016
*G. multipileum*	CWN 04670	KJ143913	–	KJ143931	KJ143972	Zhou et al. 2015
	Dai 9447	KJ143914	–	MG367588	KJ143973	Zhou et al. 2015
*G. orbiforme*	Cui 13918	MG279186	–	MG367576	MG367522	Xing et al. 2018
*G. resinaceum*	HMAS86599	AY884177	–	–	JF915435	GenBank
	CBS 194.76	KJ143916	–	KJ143934	–	Zhou et al. 2015
*G. ryvardenii*	HKAS 58053	HM138671	–	–	–	Kinge and Mih 2011
	HKAS 58054	HM138672	–	–	–	Kinge and Mih 2011
*G. sessile*	JV1209/9	KF605629	–	KJ143936	-	Li et al. 2015
	JV 1209/27	KF605630	–	KJ143937	KJ143976	Li et al. 2015
*G. sichuanense*	HMAS 42798	JQ781877	–	–	–	Li et al. 2015
	CGMCC 5.2175	KC662402	–	–	–	Yao et al. 2013
*G. sinense*	Wei5327	KF494998	KF495008	KF494976	MG367529	Xing et al. 2018
*G. tropicum*	Yuan 3490	JQ781880	–	KJ143938	–	Cao et al. 2012
	Dai 16434	MG279194	–	MG367585	MG367532	Xing et al. 2018
*G. valesiacum*	CBS428.84	JQ520218	–	–	–	Park et al. 2012
*G. williamsianum*	Dai 16809	MG279183	–	MG367588	MG367535	Xing et al. 2018
	Wei5032	KU219994	KU220024	–	–	Song et al. 2016
*G. zonatum*	FL-02	KJ143921	–	KJ143941	KJ143979	Zhou et al. 2015
	FL-03	KJ143922	–	KJ143942	KJ143980	Zhou et al. 2015
*Amauroderma rugosum*	Cui 9011	KJ531664	–	KU572504	–	Li and Yuan 2015
*Tomophagus colossus*	TC-02	KJ143923	–	KJ143943	MG367506	Zhou et al. 2015

**Figure 1.**
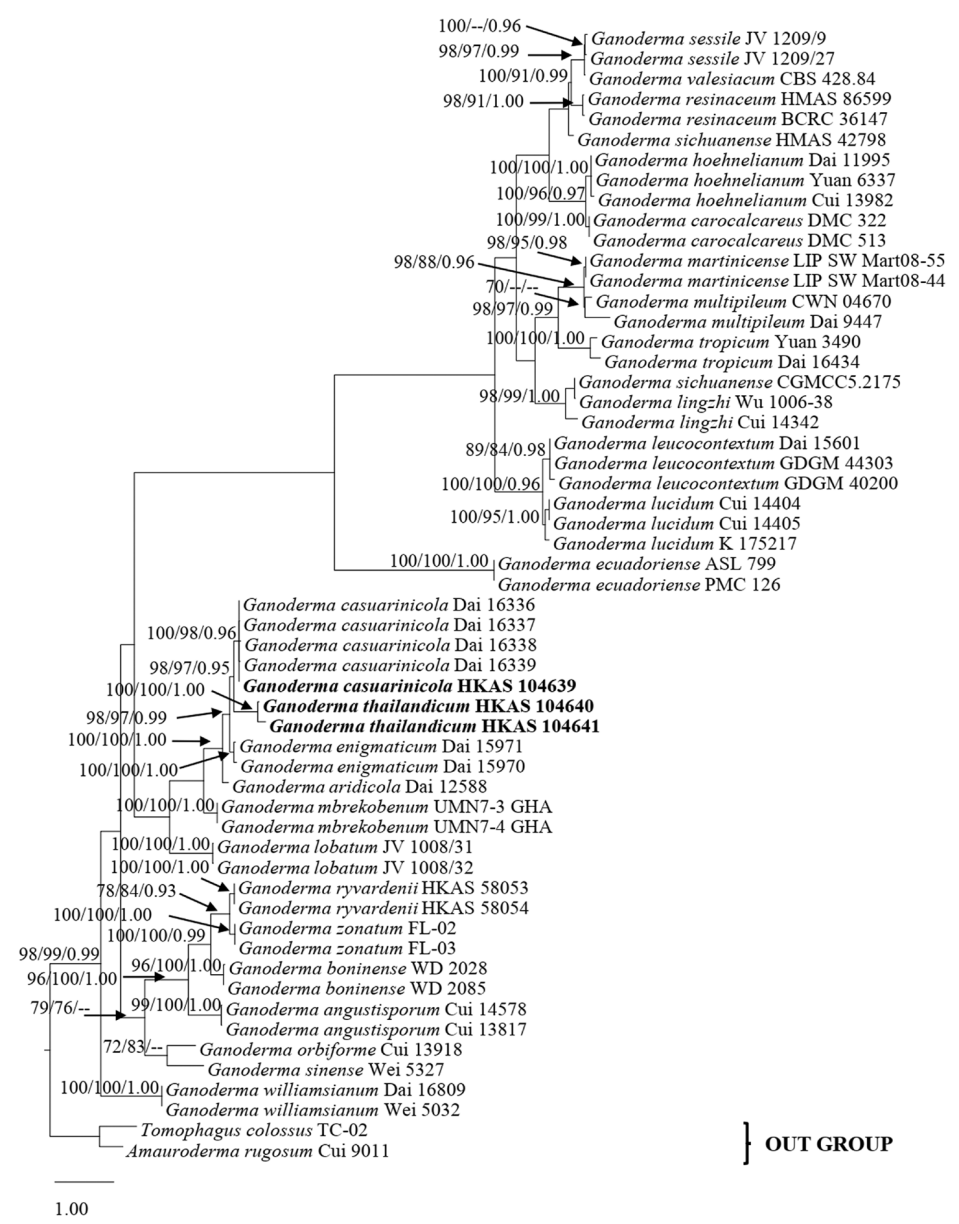
Phylogram of *Ganoderma thailandicum*, obtained from maximum likelihood (RAxML) of combined ITS, LSU, TEF1α and RPB2 datasets. Bootstrap values (BS) from maximum likelihood (ML, left) and Maximum parsimony (MP, middle) greater than 70% and Bayesian posterior probabilities (PP), greater than 0.95, are indicated above the nodes as MLBS/MPBS/PP. The tree is rooted with *Amauroderma calcitum* Cui 9011 and *Tomophagus colossus* TC-02. New species and new records are indicated in black bold.

MrModeltest v. 2.3 was used to determine the best-fitting substitution model for each single gene partition and the concatenated dataset for Bayesian analyses (Nylander 2004). Bayesian inference posterior probabilities (PP) with a GTR+I+G model was used for each partition. MrBayes v. 3.2.2 (Huelsenbeck and Ronquist 2001) was used to evaluate PP by Markov Chain Monte Carlo sampling (BMCMC) (Rannala and Yang 1996; Zhaxybayeva and Gogarten 2002). The number of generations was set at 4,000,000, with trees being sampled every 100 generations and a total of 40,000 trees obtained, resulting in an average standard deviation of split frequencies below 0.01. Based on the tracer analysis (Rambaut et al. 2014), the first 20% of trees (8,000 trees) were discarded as the burn-in phase of the analyses represented. The remaining 32,000 trees were used for calculating PP in the majority rule consensus tree (Larget and Simon 1999). ML and MP bootstrap values, equal to or greater than 70% and Bayesian Posterior Probabilities (BP) equal to or greater than 0.95 are presented above each node (Fig. 1). Trees were figured in the FigTree v. 1.4.0 programme (Rambaut 2012), edited using Microsoft Office PowerPoint 2010 and exported to Adobe Illustrator CS v. 3 (Adobe Systems, USA). Sequences derived in this study were deposited in GenBank (http://www.ncbi.nlm.nih.gov).

## Results

### Phylogenetic analyses

The phylogenetic analyses included 56 taxa (including the three new sequence data) and the tree was inferred from the combined ITS, LSU, TEF1α and RPB2 sequences, which comprise 3,360 characters with gaps; 623 characters for ITS, 930 characters for LSU, 859 characters for TEF1α and 948 characters for RPB2. The best scoring ML tree is shown in Fig. 1. Tree topologies of the ML and MP were similar to the Bayesian analysis. The dataset represents 26 *Ganoderma* species, with *Amauroderma rugosum* Cui 9011 and *Tomophagus colossus* TC-02 as the outgroup taxa. The dataset comprised 3361 total characters, of which 2378 were constant, 782 variable characters were parsimony-informative and 201 characters were parsimony-uninformative. Phylogenetic analyses indicated the placement of three isolates (HKAS 104639, HKAS 104640 and HKAS 104641) within the laccate *Ganoderma* clade. Phylogenetic results showed that the tree has two main distinct clades. The phylogenetic tree gave considerably high support for the *G. casuarinicola* strain HKAS 104639 and is closely related to the laccate *G. casuarinicola*, as well as the isolates of Guangdong, China, with good support (MLBS = 100% / MPBS = 98% / PP = 0.96), while the two newly isolated strains from this study (HKAS 104640 and HKAS 104641) formed a distinct clade (MLBS = 100% / MPBS = 100% / PP = 1.00) with a sister clade with *G. casuarinicola* clade (MLBS = 98% / MPBS = 97% / PP = 0.95).

### Taxonomy

#### Ganoderma casuarinicola

Taxon classificationFungiPolyporalesGanodermataceae

J.H. Xing, B.K. Cui & Y.C. Dai., MycoKeys 34: 93–108 (2018)

635D987A-1FC9-5AC5-8A2F-EE5B8DF149C4

Fig. 2

##### Description.

**Basidiocarps**: Substipitate to stipitate. **Pileus shape.** Annual, applanate and dimidiate when becoming mature, up to 10–16 cm in length, 4– 9 cm in width, up to 0.7–1.2 cm thick. **Pileus surface.** Distinctively zonate from the base to the margin where the new hyphae are in active development, orange, golden yellow at the base, slightly to reddish-orange, orange red, brownish-red, extended to reddish-brown, red at centre, orange to deep orange extending to the upper margin surface, with yellowish-white to pale yellow under margin surface, strongly laccate, glabrous, glossy, shiny, smooth, spathulate, shallow sulcate when fresh, thin crust overlies the pellis, thicker at the base than the margin, light in weight when dried, non-woody when dried. **Context.** Mostly yellow to light orange, orange close to crust, reddish-golden, light brown, brown near the tube layers, dense context layer but not fully homogeneous, thick near the base, tough to break when dried; generative hyphae up to 2.10–4.92 μm (*x̄* = 3.34, *n* = 50) in diam., thin walled, almost colourless, some expanded at the apex, unbranched, with clamp connections; binding hyphae 3.67–5.93 µm (*x̄* = 4.85, *n* = 50), almost colourless, thin to thick-walled, branched, with clamp connections; skeletal hyphae abundant, up to 3.49–7.34 μm (*x̄* = 5.34, *n* = 50), almost colourless, thick-walled, unbranched or with very few branches in the distal end, without clamp connections. **Hymenophore.** Trimitic, heterogeneous, up to 1.4 cm thick, generally yellow slightly to light orange, up to 4 mm thick, the lower layer (close to the tubes) on the upper layers, light brown to brown close to the tubes, presented dark brown, melanoid band. **Basidiospores.** Ellipsoid to broadly ellipsoid with double wall (ganodermoid) at maturity, yellowish brown, (*8.7*)*10.8–13.5* (*14.4*) × (*6.6*)*7.6–8.9* (*9.8*) μm (*x̄* = 12.05 × 7.8 μm, *n* = 50), with Q = 1.38 – 1.45, L = 11.68 µm, W = 8.25 µm (including myxosporium), (*7.1*)*9.9–11.2* (*12.1*) × (*5.2*)*6.7–7.3* (*8.9*) μm (*x̄* = 10.2 × 6.4 μm, *n* = 50) μm, with Q = 1.48–1.52, L = 10.65 µm, W = 7.10 µm (excluding outer myxosporium). **Tubes.** Up to 6–14 mm long, dark brown, hard, woody when dried; generative hyphae 1.0–3.7 µm in diam., occasionally with simple septa, almost colourless, thin-walled with occasionally thick walls, with clamp connections, occasionally branched; skeletal hyphae 2.7–5.1 µm in diam., thick-walled frequently branched at apex; binding hyphae 1.1–3.0 µm in diam., thin to thick-walled, frequently branched at apex. **Stipe.** Lateral, golden yellow, orange red, up to 8 cm long, 1.8 cm in diam. **Margin.** Obtuse from the substrate, soft, slippery to the touch when young, tough to break. **Pores.** Angular to round, 4–6 per mm, up to 128–195 × 148–266 µm (*x̄* = 162 × 220 μm, *n* = 50). **Pore surface.** White when fresh, turning yellowish-white to pale yellow when dry, reddish-grey when touched, greyish-brown, brownish-grey when wet. **Hyphal system.** Trimitic, generative hyphae, 2–5 µm in diam., almost colourless, thin-walled or occasionally thick-walled, with clamp connections, occasionally with irregular cuticle cells, light brown to brown in KOH; binding hyphae 3–5 µm, almost colourless, thin to thick-walled, branched, with clamp connections; skeletal hyphae abundant, up to 3–7 μm, almost colourless, thick-walled, unbranched, without clamp connections.

##### Habitat.

Solitary on *Pinus kesiya* stumps in pine forests.

##### Specimen examined.

THAILAND, Surat Thani Province, Phanom District, Khao Sok national park, 8°54'32"N, 98°31'09"E, 427 m elev., 25 June, 2018, LT2018-103 (HKAS 104639).

**Figure 2.**
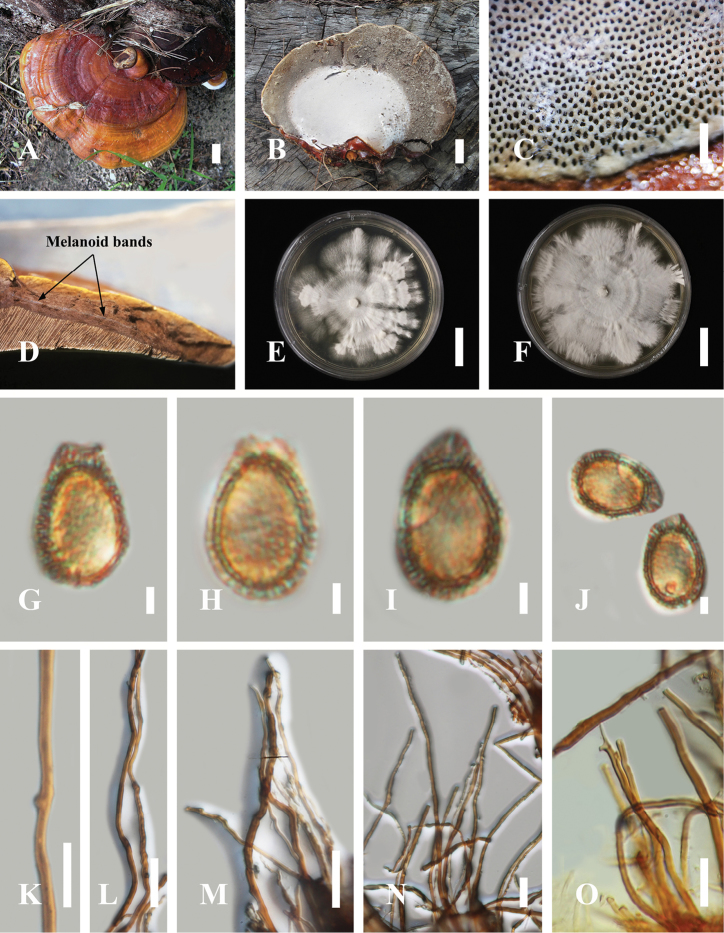
Morphology of *Ganoderma casuarinicola* (HKAS 104639) **A** The upper surface of mature basidiocarp **B** the lower surface of mature basidiocarp **C** pore characteristics **D** melanoid bands in the context tissue **E, F** culture after incubation at 25 °C for 10–14 days on Potato Dextrose Agar (PDA) **G–J** basidiospores in KOH**K** clamp connections **L** thick walled unbranched generative hyphae of context in KOH**M, N** thin-thick-walled unbranched generative and flexuous skeletal hyphae **O** thick-walled generative and skeletal hyphae of the tube layers. Scale bars: 2 cm (**A, B**); 500 µm (**C**); 2 cm (**E, F**); 2 µm (**G–J**); 5 µm (**K**); 3 µm (**L–O**).

#### Ganoderma thailandicum

Taxon classificationFungiPolyporalesGanodermataceae

T. Luangharn, P.E. Mortimer, S.C. Karunarathna & J.C. Xu sp. nov.

B2FF56D7-2287-5F91-834F-E01C886D11B6

831323

Fig. 3

##### Diagnosis.

*Ganoderma thailandicum* is characterised by its laccate deep magenta close to stipe, brownish-red at centre and light yellow of active development towards the margin on pileal surface, white pore surface, brownish-red context and absence of melanoid band.

##### Holotype.

THAILAND, Nakhon Si Thammarat Province, Khanom District, solitary on stump of *Pinus merkusii*, 10 December 2018, LT2018-105 (HKAS 104640).

##### Etymology.

The species epithet “*thailandicum*” refers to the country where the holotype was collected.

##### Description.

**Basidiocarps.** Dimidiate, laccate, substipitate to stipitate. **Pileus shape.** Annual and dimidiate when mature, up to 3–9 cm in length, 3–6 cm in width, up to 0.4–1.8 cm thick at centre of pileus close to the stipe, obtuse from the substrate. **Pileus surface.** Laccate, glabrous, glossy, smooth, soft, umbonate, distinctly concentrically zonate, greyish-magenta to deep magenta at stipe, greyish-ruby, greyish-red to brownish-red at centre, extended to reddish-orange to slightly pale red with light yellow to vivid yellow of active development towards the margin, thin crust overlaying the pileus, sometimes convex sulcate extending at centre, with distinct concentric zones, with fine furrows at centre extended to the margin, thicker at the base than the margin, consistency hard when young to mature, some cracked when old, non-woody, light in weight when dried. **Hymenophore.** Trimitic, up to 0.4–2.4 cm thick, heterogeneous with greyish-red close to the upper layers slightly to brownish-red to reddish-brown close to the tubes. **Context**. Mostly brownish-red to reddish-brown in Melzer’s reagent, absent of melanoid band, with dense context layer. **Basidiospores.** Ellipsoid to broadly ellipsoid with some globose with double wall (ganodermoid) at maturity, light brown to reddish-brown in Congo red reagent, (*6.8*)*8.4–9.7* (*10.2*) × (*5.8*)*6.5–7.3* (*7.7*) μm (*x̄* = 9.1 × 6.9 μm, *n* = 50), with Q = 1.29–1.35, L = 9.13 µm, W = 6.96 µm (including myxosporium), (*5.4*)*7.6–9.6* (*10.0*) × (*4.7*)*5.8–6.9* (*7.4*) μm (*x̄* = 7.6 × 6.0 μm, *n* = 50) μm, with Q = 1.32–1.38, L = 8.64 µm, W = 6.42 µm (excluding outer myxosporium). **Tubes.** Up to 0.5 mm close to margin to 7 mm at centre in length, brown to dark brown, hard, woody when dried; generative hyphae 2.73–4.74 µm in diam., almost colourless, thin-walled with occasionally thick walls, with clamp connections, occasionally branched; skeletal hyphae 3.76–5.81 µm in diam., thick-walled frequently branched at apex; binding hyphae 3.24–5.84 µm in diam., thin to thick-walled, frequently branched at apex. **Stipe.** Lateral, pale red to vivid red, greyish-red to red when present, with violet brown when mature, different from and darker than pileus, up to 3–5 cm long, 2.5–3.0 cm in diam., 1.8–2.7 cm thick. **Margin.** Up to 0.4–0.8 cm thick when becoming mature, active growing margin white on the upper and under margin surface when fresh, with a yellow line under the pileus, round, soft, smooth, slippery when touched when young to mature stage, without any zonation, tough when broken. **Pores.** Angular to round, 4–8 per mm, up to 121–176 × 174–247 µm (*x̄* = 155 × 209 μm, *n* = 50). **Pore surface.** White when fresh, grey at centre, slightly orange grey at margin, brownish-grey when touched, turning brownish-orange when dry, grey when wet. **Hyphal system.** Trimitic, light orange to deep orange, reddish-brown in Melzer’s reagent; generative hyphae, 2.65–4.58 µm (*x̄* = 3.82, *n* = 50) in diam., almost colourless, mostly thick-walled, occasionally thin-walled, bearing clamp connections, occasionally with irregular cuticle cells; binding hyphae 3.32–6.28 µm (*x̄* = 5.53, *n* = 50), almost colourless, thin-walled, occasionally branched in the distal end, with clamp connections; skeletal hyphae abundant, up to 3.40–6.78 μm (*x̄* = 5.73, *n* = 50), almost colourless, thick-walled and unbranched. **Context**. Mostly brownish-red in Melzer’s reagent, reddish-brown, with greyish-red close to crust, dense context layer, agglutinate mass, usually solid in basal part, thick near the base, tough to break when dried; generative hyphae up to 2.80–5.75 μm (*x̄* = 4.36, *n* = 50) in diam., mostly colourless, thick-walled, with clamp connections, occasionally with simple septa; binding hyphae 1.23–4.75 µm (*x̄* = 2.49, *n* = 50), colourless, thin-walled or with a very few branches in the distal end, with clamp connections; abundant skeletal hyphae up to 3.30–7.51 μm (*x̄* = 5.75, *n* = 50), almost colourless, thick-walled, unbranched, with clamp connections and occasionally with simple septa. **Cuticle cells.** Clavate to narrowly clavate, tuberculate, occasionally with irregular cuticle cells, mostly thick-walled, occasionally thin-walled with simple septa. **Basidia.** Clavate, with 4 sterigmata, 12.2–19.6 × 8.3–10.9 µm, light brown (5D6) to yellowish in Melzer’s reagent.

##### Material examined.

THAILAND, Nakhon Si Thammarat Province, Khanom District, solitary on stump of *Pinus merkusii*, 11°45'58"N, 99°47'43"E, 499 m elev., 10 December 2018, LT2018-105 and LT2018-106, specimens no. HKAS 104640 and HKAS 104641.

**Figure 3.**
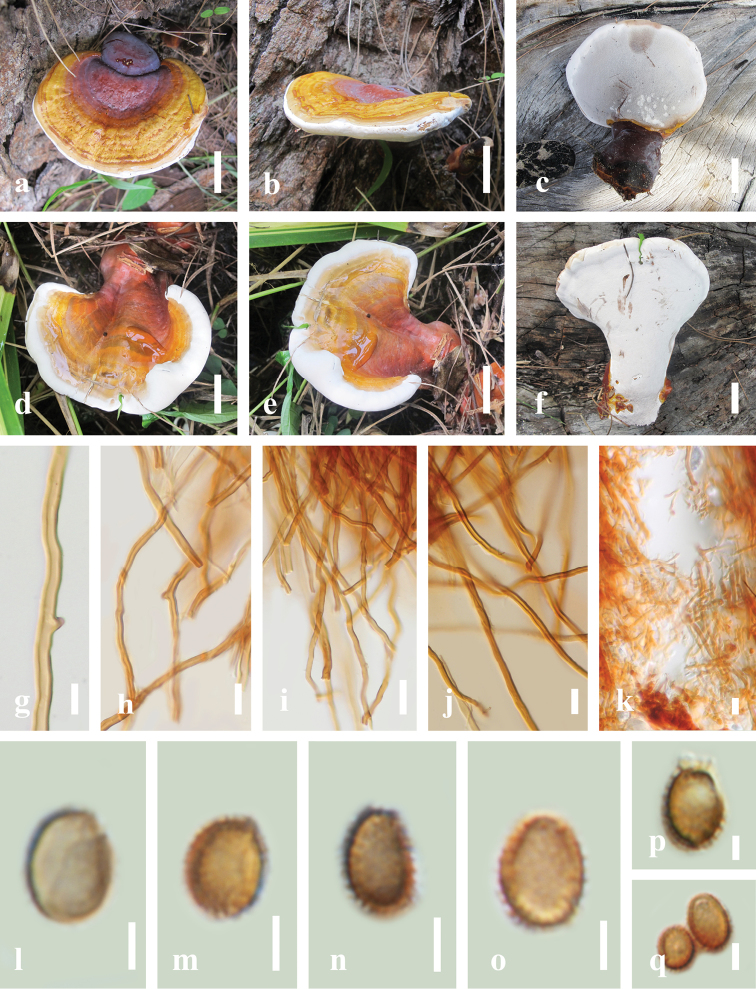
Morphological characteristics of *Ganoderma thailandicum* (HKAS 104640, HKAS 104641). **A, B** Mature basidiocarps (HKAS 104640) **C** lower surface of mature basidiocarp (HKAS 104640) **D, E** development of young to mature fruiting bodies (HKAS 104641) **F** lower surface (HKAS 104641) **G** clamp connections **H** thick-walled unbranched generative hyphae with clamp connections of context in KOH**I** thick-walled, skeletal hyphae in KOH without septa **J** thick-walled sparingly branched skeletal hyphae in Melzer’s reagent **K** hyphae of tube layers **L–Q** basidiospores in 3% Congo red reagent. Scale bars: 2 cm (**A–F**); 10 µm (**G**); 15 µm (**H–K**); 3 µm (**L–P**); 5 µm (**Q**).

## Discussion

In this study, we describe a new species of *Ganoderma* growing on *Pinus* sp. in tropical southern Thailand, in a well-researched genus. This is not surprising as Hyde et al. (2018) found that up to 96% of species discovered in northern Thailand were new to science. *Ganoderma casuarinicola* was collected on a *Pinus kesiya* stump in a pine forest at Surat Thani Province in Thailand, while two collections of *Ganoderma thailandicum* were collected on *Pinus merkusii* stumps from Kanom District, Nakhon Si Thammarat Province in Thailand. All three collections grouped as sister taxa to the laccate *Ganoderma* clade, their morphological characteristics and molecular analyses providing insights to resolve species delimitation. In this study, we introduce *G. casuarinicola* (HKAS 104639) as a new record to Thailand which grouped with the holotype from Guangdong, China (Fig. 1) with high statistical support (MLBS = 100% / MPBS = 98% / PP = 0.96) and *G. thailandicum* is described as a new species, the two collections of *G. thailandicum* (HKAS 104640 and HKAS 104641) grouping together as a distinct clade with 100% ML, 100% MP and 1.00 PP support.

Our findings are consistent with Xing et al. (2018), who demonstrated that *G. casuarinicola* forms a sister clade with *G. aridicola* J.H. Xing & B.K. Cui, from South Africa and *G. enigmaticum* M.P.A. Coetzee, Marinc., M.J. Wingf., from Africa (Coetzee et al. 2015). The morphological differences of these three *Ganoderma* species were detailed in Xing et al. (2018). Moreover, our study allows us to compare the holotypes of *G. casuarinicola* from Guangdong and our collection from Thailand. The Guangdong’s *G. casuarinicola* shows its distinctive sectorial to shell-shaped, 10 cm long and 7 cm wide pileus (Xing et al. 2018), while the Thai *G. casuarinicola* shows its annual, applanate to dimidiate shape, 3–16 cm long and 1.5–3 cm wide pileus, larger than the Guangdong collection. Our *G. casuarinicola* collections show longer tubes of 6–14 mm, while the tubes of the Guangdong collection are 9 mm long; however, our collections show a thinner margin (0.8–1.2 cm thick) than the Guangdong collection (2 cm thick) (Xing et al. 2018). Macro-morphological characteristics of our *G. casuarinicola* share similarities with the holotype collection, such as strongly laccate, shallow sulcate, reddish-brown pileus surface, lateral stipe shape, white pore surface and brown context.

Micro-morphological characteristics of the context layers of both Guangdong and Thai *G. casuarinicola* share similar characteristics, such as the dense light brown to brown context layers; thin to thick-walled generative hyphae; thin-walled binding hyphae; and thick-walled skeletal hyphae. Although both type specimens and our collection of *G. casuarinicola* collection have mostly distinctive yellowish-brown basidiospores, Thai *G. casuarinicola* collections have a smaller size range of (*8.7*)*10.8–13.5* (*14.4*) × (*6.6*)*7.6–8.9* (*9.8*) μm than the type of *G. casuarinicola* (*8.3*–)*9.0–10.2* (–*11.5*) × (*4.5*–)5.*0–6.0* (–*7.0*) µm (including myxosporium). However, the type of *G. casuarinicola* does not have the melanoid band (Xing et al. 2018), while our collection has a dark brown, melanoid band. Although both type specimens and our *G. casuarinicola* collections are grouped in the same clade, macro-morphologically, their pilei are very different, most probably due to geographical and climatic changes. Boddy et al. (2014) also mentioned that climate change and geography affect fungi in many ways, especially regarding phenological changes of fungal fruiting and the spatial and temporal distribution of hosts.

According to our phylogenetic analyses (Fig. 1), collections of *G. thailandicum* were grouped as a sister to *G. aridicola*, *G. casuarinicola*, and *G. enigmaticum* as a well-supported clade of 100% ML, 100% MP and 1.00 PP statistical supports. *Ganoderma aridicola*, *G. casuarinicola*, *G. enigmaticum* and *G. thailandicum* share morphological similarities of laccate to strong laccate upper pileus surface and ellipsoid to broadly ellipsoid basidiospores at maturity. *Ganoderma aridicola* (Xing et al. 2016), *G. casuarinicola* (Xing et al. 2018) and *G. enigmaticum* (Coetzee et al. 2015) are considered as members of the *G. lucidum* complex and our *G. thailandicum* is also clustered within the *G. lucidum* complex, according to the results of the phylogenetic analyses. Our phylogenetic tree showed *G. thailandicum* clustered together with *G. casuarinicola*. Although *G. thailandicum* and *G. casuarinicola* form a distinctive laccate pileus surface, their macro- and micro-morphological characteristics are quite different. *Ganoderma thailandicum* can be easily distinguished from *G. casuarinicola*, by its deep magenta colour near the stipe, brownish-red colour at the centre of the pileus surface and vivid yellow colour at the actively-developed margin, while the fruiting bodies of *G. casuarinicola* are homogenously brownish-red to reddish-brown at maturity. *Ganoderma thailandicum* also has a smaller sized pileus (3–9 cm long, 3–6 cm width, 0.4–1.8 cm thick), while *G. casuarinicola* has a larger pileus (up to 10 cm long, 4–9 cm width, up to 2 cm thick). *Ganoderma thailandicum* has a smaller pore size (4–8 per mm) than *G. casuarinicola* (4–6 per mm) and *G. thailandicum* has narrower basidiospores (6.93 × 9.11 μm; including myxosporium) than *G. casuarinicola* (8.25 × 11.68 μm; including myxosporium). The basidiopore shapes of *G. thailandicum* are distinctive, with ellipsoid to broadly ellipsoid or some globose, while basidiospores of *G. casuarinicola* are mostly ellipsoid to broadly ellipsoid at maturity. Both *G. thailandicum* and *G. casuarinicola* are quite similar by having angular to round pore shapes. The differences of *G. aridicola* and *G. enigmaticum* have been described in Xing et al. (2016). *Ganoderma mbrekobenum* can be differentiated from *G. casuarinicola* and *G. thailandicum* by its woody to corky texture when dried, with dimitic hyphal system, ovoid and bitunicate basidiospores (Crous et al. 2016).

*Casuarina* has been reported as a host genus for *G. casuarinicola* (Xing et al. 2018), which is found in coastal areas, while our *G. casuarinicola* collection was found on dead *Pinus kesiya* wood, thus this is the first *Pinus* host recorded for *G. casuarinicola*. Based on comprehensive morphological characteristics and molecular analyses, we report *G. casuarinicola* as a new record to Thailand, with *G. thailandicum* as a new species from Thailand.

## Supplementary Material

XML Treatment for Ganoderma casuarinicola

XML Treatment for Ganoderma thailandicum
